# Development and internal validation of a nomogram for predicting lymph node metastasis posterior to the right recurrent laryngeal nerve in papillary thyroid carcinoma: a single-center retrospective study

**DOI:** 10.3389/fendo.2026.1953516

**Published:** 2026-09-17

**Authors:** Qiran Lu, Luxin Wei, Lifeng Zhang

**Affiliations:** Department of Thyroid and Breast Surgery, The Fourth Affiliated Hospital of Soochow University, Suzhou, Jiangsu, China

**Keywords:** central lymph node metastasis, decision curve analysis, lateral lymph node metastasis, LNprRLN, lymph nodes posterior to the right recurrent laryngeal nerve, nomogram, papillary thyroid carcinoma, risk prediction

## Abstract

**Background:**

The lymph nodes posterior to the right recurrent laryngeal nerve (LN-prRLN) lie deep in the right central compartment. Clearing them is technically demanding and adds to the risk of injury to the nerve and parathyroid glands, whereas occult metastases left behind may cause persistent or recurrent disease. Knowing in advance which patients are likely to have LN-prRLN metastasis would help tailor the extent of central neck dissection.

**Methods:**

We retrospectively reviewed 320 patients with papillary thyroid carcinoma (PTC) treated at a single tertiary center between August 2021 and February 2026, all of whom underwent thyroidectomy with right central neck dissection that included separate LN-prRLN harvesting. Candidate clinicopathological variables were screened by univariable logistic regression; non-redundant binary variables with P<0.05 then entered a multivariable Firth penalized-likelihood model, used because a single event among the 166 central-node-negative patients biased the ordinary maximum-likelihood estimate. The independent predictors formed a nomogram, internally validated by 1,000 bootstrap resamples that repeated the entire model-building procedure, and was assessed for discrimination, calibration (including the optimism-corrected calibration slope and the Hosmer–Lemeshow test) and clinical utility (decision curve analysis, DCA).

**Results:**

LN-prRLN metastasis was present in 51 of 320 patients (15.9%). Multifocality (odds ratio [OR] 4.46), tumor diameter >10 mm (OR 2.87), isthmic/near-isthmic location (OR 6.28), central lymph node metastasis (OR 49.15) and lateral lymph node metastasis (OR 13.90) were independent predictors (all P<0.05), whereas capsular invasion was not retained (P = 0.286). The resulting nomogram reached an area under the receiver operating characteristic curve of 0.936 (95% confidence interval 0.901–0.971; optimism-corrected 0.927), with a sensitivity of 0.843 and a specificity of 0.896 at the optimal cutoff of 0.226. Calibration curves lay close to the ideal line (optimism-corrected calibration slope 0.961, intercept −0.024; Hosmer–Lemeshow P = 0.285), and DCA showed a positive net benefit across the clinically plausible range of threshold probabilities.

**Conclusion:**

A nomogram combining multifocality, tumor size, isthmic location, and central and lateral compartment metastasis predicted LN-prRLN metastasis with high discrimination and acceptable internal calibration. Intended for use with preoperative, imaging-based assessment of nodal status, the nomogram requires external validation, ideally multicenter and prospective, before routine use. It should be emphasized, however, that central and lateral nodal status — the two strongest predictors — were coded from the final histopathological report, and because the sensitivity of preoperative ultrasound and contrast-enhanced computed tomography for central compartment metastasis is limited, the performance reported here is an upper bound on what can be expected when the nomogram is used with imaging-based inputs.

## Introduction

Thyroid carcinoma is one of the most common endocrine malignancies, and its incidence has risen markedly over the past few decades (1). Papillary thyroid carcinoma (PTC) accounts for the large majority of cases and usually has a favorable prognosis, but cervical lymph node metastasis is frequent, occurs early, and predisposes to locoregional recurrence and reoperation (2, 3). For most PTCs the central compartment (level VI) is the first echelon of nodal spread, which is why central neck dissection occupies such a prominent place in surgical management (2).

One subregion of the right central compartment deserves particular attention: the lymph nodes lying posterior to the right recurrent laryngeal nerve (LN-prRLN), often called the posterior right paratracheal nodes. Because the subdivision of level VI into VIa and VIb subregions is not standardized across nodal-level classifications, we use purely descriptive terms throughout: LN-prRLN for the nodal tissue lying dorsal to the right RLN, and superficial central nodes for the remaining level VI nodes, that is, the prelaryngeal, pretracheal and paratracheal nodes lying anterior to the nerve. On the right side, the recurrent laryngeal nerve (RLN) loops around the subclavian artery and then runs obliquely from lateral to medial, leaving a deep triangular space behind it; the left RLN follows a more vertical, paratracheal course and has no comparable deep nodal recess (4, 5). Reported rates of LN-prRLN metastasis vary widely, from about 2.7% to 38.3%, and this location is a recognized reservoir of occult disease (4, 6, 7).

A number of studies and meta-analyses have linked LN-prRLN metastasis to tumor size, multifocality, capsular or extrathyroidal invasion, and metastasis in the superficial central and lateral compartments (6–8). Several groups have gone further and built risk models for this specific subregion. Luo et al. derived the first such model, from age, tumor size, capsular invasion and right lateral nodal metastasis, with an area under the curve (AUC) of 0.790 in the derivation set and 0.878 in a randomly split validation set (9). Zhu et al. combined preoperative features with intraoperative frozen-section results for the prelaryngeal, pretracheal and paratracheal node groups and reached a concordance index of 0.87 (10). Gong et al. added conventional and contrast-enhanced ultrasound characteristics of the primary tumor (4), and Deng et al. recently derived a model driven mainly by the number of metastatic prelaryngeal and paratracheal nodes in clinically node-negative patients (11). A nomogram turns such a multivariable model into a simple graphical calculator and is now widely used for individualized risk prediction in thyroid cancer (12, 13). What these models do not yet provide is agreement, and they cannot readily be validated against one another. They share almost no predictors; more practically, each depends on inputs that are not recorded in every center — metastatic node counts by nodal subgroup (10, 11), intraoperative frozen-section results for individual node groups (10), or contrast-enhanced ultrasound characteristics (4) — so that externally validating any of them requires data that a retrospective cohort such as ours simply does not contain. We considered testing an existing model in our cohort, but none could be reconstructed from routinely documented variables. Moreover, none of these models has been validated outside the cohort in which it was built, so it remains unknown which routinely available variables reproducibly identify patients at risk. The gap in the literature is therefore twofold: a model restricted to variables documented in every standard thyroid cancer work-up, and independent cohorts in which the recurring candidate predictors can be re-examined. We accordingly examined the factors associated with LN-prRLN metastasis in 320 patients with PTC and developed and internally validated a nomogram built from five variables that are documented in virtually every thyroid cancer work-up, intended to support case-by-case decisions about LN-prRLN dissection.

## Materials and methods

### Study design and patients

This single-center retrospective study was conducted at the Department of Thyroid and Breast Surgery, The Fourth Affiliated Hospital of Soochow University. We reviewed consecutive patients who underwent thyroidectomy with right-sided central neck dissection, including separate harvesting of the LN-prRLN, between August 2021 and February 2026. At our center, separate harvesting of the LN-prRLN is a routine component of every right-sided central neck dissection performed for PTC, irrespective of preoperative or intraoperative suspicion of nodal metastasis; no patients were selected for LN-prRLN dissection on the basis of any clinical or pathological feature. The cohort is therefore a consecutive, otherwise unselected series of eligible patients undergoing this procedure during the study period. Inclusion criteria were: (i) histopathologically confirmed PTC; and (ii) LN-prRLN tissue that was dissected and separately submitted for pathological evaluation. Exclusion criteria were: (i) other thyroid malignancies or coexisting non-thyroidal malignancy; (ii) previous thyroid surgery or neck irradiation; and (iii) incomplete clinicopathological or nodal data. After applying these criteria, 320 patients were included in the final analysis.

The study was performed in accordance with the Declaration of Helsinki and was approved by the Ethics Committee of The Fourth Affiliated Hospital of Soochow University (approval No. 2026LYP260239). Given the retrospective design and the use of de-identified data, the requirement for written informed consent was waived by the ethics committee.

### Surgical procedure and definition of LN-prRLN

All operations were performed by experienced thyroid surgeons. As routine institutional practice, all patients with thyroid cancer underwent both preoperative cervical ultrasonography and contrast-enhanced computed tomography (CECT) of the neck. The extent of thyroidectomy and neck dissection was determined according to this dual-modality imaging assessment, intraoperative findings and prevailing guidelines (2). The right RLN was routinely identified and preserved along its cervical course. The LN-prRLN was defined as the fibrofatty lymphatic tissue situated dorsal to the right RLN, bounded superiorly by the point at which the nerve enters the larynx, inferiorly by the level of the right subclavian artery, and posteriorly by the prevertebral fascia. After the RLN was fully exposed, the nodal tissue posterior to the nerve was dissected separately, labeled as LN-prRLN, and sent for independent histopathological examination. LN-prRLN metastasis was defined as the pathological confirmation of at least one metastatic node within this specimen.

### Variables and outcome

The following variables were retrieved from medical and pathological records: age, sex, tumor focality (unifocal vs. multifocal), maximum tumor diameter, primary tumor location (upper pole, middle, lower pole, or isthmus/near-isthmus), coexisting Hashimoto thyroiditis, capsular invasion, central lymph node metastasis (defined as metastasis in the central compartment exclusive of the LN-prRLN), and lateral lymph node metastasis. All candidate variables, including central and lateral lymph node status, were coded from the final histopathological report and therefore represent the postoperative reference standard rather than preoperative imaging or intraoperative findings; no frozen-section data were used. How each predictor is approximated at the time of surgical decision-making is addressed in the Discussion. For analysis, three pre-specified binary variables were derived: age <45 years, maximum tumor diameter >10 mm, and isthmic/near-isthmic location versus all other locations. The 45-year cutoff was pre-specified because it was the age threshold used to stratify prognostic risk in the American Joint Committee on Cancer/Union for International Cancer Control (AJCC/UICC) tumor-node-metastasis (TNM) staging system for differentiated thyroid carcinoma up to the 7th edition (14) and has since been widely adopted for dichotomizing age in studies of thyroid-cancer nodal metastasis, which aids comparability with earlier work; the 55-year threshold introduced in the 8th edition was examined in a sensitivity analysis. The primary outcome was the presence of LN-prRLN metastasis.

### Statistical analysis

All analyses were performed in R 4.4.2. Firth penalized-likelihood logistic regression, together with its profile penalized-likelihood confidence intervals and penalized likelihood-ratio tests, was fitted with the logistf package; receiver operating characteristic (ROC) curves, their DeLong confidence intervals and DeLong tests for correlated curves were obtained with pROC 1.18.5; the nomogram and the calibration curves were produced with rms 6.8-1, the linear predictor of the penalized model being supplied to rms as a fixed offset so that the plotted model is exactly the model reported; and decision curve analysis was performed with the dcurves package. The full analysis script is provided as supplementary material. Continuous variables are presented as mean ± standard deviation and were compared with the independent-samples t-test, with the Mann–Whitney U test used as a distribution-free check; the two tests agreed for every variable and the t-test values are those tabulated. Categorical variables are presented as counts (percentages) and were compared with the chi-square test with Yates continuity correction. All study variables were taken from standardized operative notes and synoptic pathology reports in which these fields are mandatory, so the dataset contained no missing values, and no imputation was performed.

The sample size available for model development was assessed against the criteria of Riley et al. (15). With five predictor parameters, an outcome prevalence of 15.9% and an observed Cox–Snell R² of 0.36, the minimum sample size required was 98 patients for an expected shrinkage factor of 0.9, 136 for an expected optimism in R² below 0.05, and 206 for estimating the overall outcome risk with a margin of error of 0.05. The available 320 patients with 51 events, corresponding to 10.2 events per predictor in the final model and 8.5 in the six-variable model, therefore exceeded all three requirements, although the margin over the most demanding criterion is modest.

Each candidate variable was tested against LN-prRLN metastasis by univariable binary logistic regression, with the overall effect of the four-category location variable assessed by a likelihood-ratio test. Variables that encoded the same information in different ways were not entered together. The non-redundant binary variables that reached P<0.05, namely multifocality, tumor diameter >10 mm, isthmic/near-isthmic location, capsular invasion, central lymph node metastasis and lateral lymph node metastasis, were then entered together into a multivariable Firth penalized-likelihood logistic regression model with the Jeffreys-prior penalty (16, 17). Firth’s method was chosen because the data are sparse rather than separated. Only one of the 166 patients without central lymph node metastasis had LN-prRLN metastasis, so although ordinary maximum likelihood did converge to finite coefficients, the estimate for this variable carried the upward small-sample bias and the extreme imprecision expected of a cell containing a single event, and penalization removed most of that bias. Complete and quasi-complete separation were formally excluded by verifying that all coefficients remained finite and that no fitted probability reached 0 or 1. We report regression coefficients (β), standard errors (SE), adjusted odds ratios (OR), 95% confidence intervals (CI) obtained by profile penalized likelihood, and two-sided P values from penalized likelihood-ratio tests. Because the intervals and the P values are derived from the same profile penalized likelihood, they are mutually consistent, and an interval that excludes an OR of 1 always corresponds to P<0.05. Collinearity among the retained predictors was assessed by variance inflation factors.

Variables that remained statistically significant (P<0.05) in the multivariable model were retained. After capsular invasion was excluded, the model was refitted by the same penalized-likelihood method in the five remaining variables, and this final model was used to construct a nomogram. Model discrimination was quantified by the area under the ROC curve (AUC), whose 95% CI was obtained by DeLong’s method (18). The AUC of the combined model was compared with the AUC of each individual predictor by DeLong’s test for two correlated ROC curves estimated in the same patients. The optimal probability threshold was chosen by the maximum Youden index; sensitivity, specificity and predictive values are reported at that threshold, together with a high-sensitivity operating point that reflects the asymmetric cost of leaving an involved node behind.

The model was internally validated with 1,000 bootstrap resamples (19). So that the resulting optimism estimate would capture the variable-selection step and not merely coefficient re-estimation, the entire model-building procedure was repeated in full within every bootstrap sample: univariable screening at P<0.05, the multivariable Firth penalized-likelihood fit, and backward removal of any predictor that was non-significant on penalized likelihood-ratio testing. In each resample the refitted model was evaluated on that bootstrap sample (apparent performance) and on the original cohort (test performance). The mean difference between the two defined the optimism, which was subtracted from the apparent values to yield the optimism-corrected C-statistic, calibration slope and calibration intercept. How often each candidate variable survived the full selection procedure across the resamples was recorded as a measure of selection stability. Calibration was examined further with apparent and bias-corrected calibration curves (20) and with the Hosmer–Lemeshow goodness-of-fit test. The latter is reported for completeness only: with 51 events and only 27 distinct predicted probabilities, the grouping the test requires is not unique and the statistic is sensitive to how tied predictions are allocated to bins. The clinical utility of the model was assessed by decision curve analysis (DCA), which plots net benefit against the high-risk threshold probability, and net benefit differences were converted into the number of unnecessary dissections avoided per 100 patients at equal detection (21, 22). Reporting followed the Transparent Reporting of a multivariable prediction model for Individual Prognosis Or Diagnosis (TRIPOD) recommendations (23). A two-sided P value <0.05 was considered statistically significant.

Three sensitivity analyses were specified in advance. First, the 45-year age dichotomy was replaced by the 55-year threshold of the 8th-edition staging system, and age and sex were each added back to the final model. Second, maximum tumor diameter was entered as a continuous variable in place of the >10 mm dichotomy. Third, the model was refitted with central nodal status removed, to quantify how much of its performance depends on that single variable.

## Results

### Patient characteristics

A total of 320 patients with PTC were analyzed, of whom 51 (15.9%) had pathologically confirmed LN-prRLN metastasis and 269 (84.1%) did not. The cohort comprised 104 men (32.5%) and 216 women (67.5%), with a mean age of 40.4 ± 12.1 years; 151 patients (47.2%) had multifocal disease, 127 (39.7%) had a tumor larger than 10 mm, and 33 (10.3%) had an isthmic/near-isthmic primary tumor. Central and lateral lymph node metastasis were present in 154 (48.1%) and 48 (15.0%) patients, respectively.

Compared with patients without LN-prRLN metastasis, those with metastasis had significantly larger tumors (1.45 ± 0.77 vs. 1.05 ± 0.69 cm; P<0.001) and higher proportions of tumor diameter >10 mm (66.7% vs. 34.6%; P<0.001), multifocality (74.5% vs. 42.0%; P<0.001), isthmic/near-isthmic location (21.6% vs. 8.2%; overall location P = 0.024), capsular invasion (31.4% vs. 15.6%; P = 0.013), central lymph node metastasis (98.0% vs. 38.7%; P<0.001) and lateral lymph node metastasis (52.9% vs. 7.8%; P<0.001). Age, sex and coexisting Hashimoto thyroiditis did not differ significantly between the two groups (all P>0.05). The baseline characteristics are summarized in **Table 1**.

**Table 1:** Baseline characteristics of patients with papillary thyroid carcinoma according to LN-prRLN metastasis status.

Characteristic	Total (n=320)	LN-prRLN + (n=51)	LN-prRLN − (n=269)	P value
Age, years (mean ± SD)	40.4 ± 12.1	40.8 ± 10.9	40.3 ± 12.4	0.798
Age <45 years, n (%)	227 (70.9)	37 (72.5)	190 (70.6)	0.914
Male sex, n (%)	104 (32.5)	20 (39.2)	84 (31.2)	0.340
Tumor size, cm (mean ± SD)	1.11 ± 0.71	1.45 ± 0.77	1.05 ± 0.69	<0.001
Tumor diameter >10 mm, n (%)	127 (39.7)	34 (66.7)	93 (34.6)	<0.001
Multifocality, n (%)	151 (47.2)	38 (74.5)	113 (42.0)	<0.001
Isthmic/near-isthmic location, n (%)	33 (10.3)	11 (21.6)	22 (8.2)	0.024*
Hashimoto thyroiditis, n (%)	58 (18.1)	8 (15.7)	50 (18.6)	0.768
Capsular invasion, n (%)	58 (18.1)	16 (31.4)	42 (15.6)	0.013
Central LN metastasis, n (%)	154 (48.1)	50 (98.0)	104 (38.7)	<0.001
Lateral LN metastasis, n (%)	48 (15.0)	27 (52.9)	21 (7.8)	<0.001

SD, standard deviation; LN, lymph node. *P value for the overall four-category location variable (chi-square test). Continuous variables were compared with the independent-samples t-test; categorical variables with the chi-square test with Yates continuity correction.

### Univariable analysis

In univariable logistic regression (**Table 2**), multifocality (OR 4.04, 95% CI 2.06–7.92; P<0.001), maximum tumor diameter as a continuous variable (OR 1.95 per 1 cm, 95% CI 1.35–2.84; P<0.001), tumor diameter >10 mm (OR 3.78, 95% CI 2.01–7.14; P<0.001), isthmic/near-isthmic location versus other locations (OR 3.09, 95% CI 1.39–6.85; P = 0.006; overall location likelihood-ratio P = 0.042), capsular invasion (OR 2.47, 95% CI 1.26–4.86; P = 0.009), central lymph node metastasis (OR 79.33, 95% CI 10.79–583.01; P<0.001) and lateral lymph node metastasis (OR 13.29, 95% CI 6.55–26.96; P<0.001) were significantly associated with LN-prRLN metastasis. Age (whether modeled continuously or dichotomized at 45 years), sex and Hashimoto thyroiditis were not significantly associated with the outcome (all P>0.05).

**Table 2:** Univariable logistic regression analysis of factors associated with LN-prRLN metastasis.

Variable	OR	95% CI	P value
Age (per 1 year)	1.00	0.98–1.03	0.797
Male sex (vs. female)	1.42	0.77–2.63	0.266
Multifocality (vs. unifocal)	4.04	2.06–7.92	<0.001
Tumor size (per 1 cm)	1.95	1.35–2.84	<0.001
Tumor diameter >10 mm (vs. ≤10 mm)	3.78	2.01–7.14	<0.001
Isthmic/near-isthmic (vs. other location)	3.09	1.39–6.85	0.006
Hashimoto thyroiditis (vs. no)	0.81	0.36–1.84	0.622
Capsular invasion (vs. no)	2.47	1.26–4.86	0.009
Central LN metastasis (vs. no)	79.33	10.79–583.01	<0.001
Lateral LN metastasis (vs. no)	13.29	6.55–26.96	<0.001

OR, odds ratio; CI, confidence interval; LN, lymph node. The overall likelihood-ratio P value for the four-category tumor location variable was 0.042.

### Multivariable analysis and nomogram construction

The six non-redundant binary variables with univariable P<0.05 (multifocality, tumor diameter >10 mm, isthmic/near-isthmic location, capsular invasion, central lymph node metastasis and lateral lymph node metastasis) were entered together into a multivariable Firth penalized-likelihood logistic regression model (**Table 3**), fitted by penalized likelihood because of the sparse central-node-negative stratum described in the Methods. Multifocality (OR 4.19, 95% CI 1.77–10.68; P<0.001), tumor diameter >10 mm (OR 2.68, 95% CI 1.17–6.39; P = 0.020), isthmic/near-isthmic location (OR 5.69, 95% CI 1.76–19.29; P = 0.004), central lymph node metastasis (OR 44.52, 95% CI 10.59–426.16; P<0.001) and lateral lymph node metastasis (OR 14.28, 95% CI 5.57–40.59; P<0.001) remained independent predictors, whereas capsular invasion was no longer significant (OR 1.74, 95% CI 0.62–4.74; P = 0.286).

**Table 3:** Multivariable logistic regression analysis of factors associated with LN-prRLN metastasis.

Variable	β	SE	OR	95% CI	P value
Multifocality	1.432	0.452	4.19	1.77–10.68	<0.001
Tumor diameter >10 mm	0.987	0.429	2.68	1.17–6.39	0.020
Isthmic/near-isthmic location	1.739	0.605	5.69	1.76–19.29	0.004
Capsular invasion	0.554	0.514	1.74	0.62–4.74	0.286
Central LN metastasis	3.796	0.860	44.52	10.59–426.16	<0.001
Lateral LN metastasis	2.659	0.503	14.28	5.57–40.59	<0.001
Final five-variable model (used to build the nomogram)					
Multifocality	1.495	0.450	4.46	1.90–11.32	<0.001
Tumor diameter >10 mm	1.053	0.429	2.87	1.25–6.83	0.013
Isthmic/near-isthmic location	1.837	0.600	6.28	1.97–21.20	0.002
Central LN metastasis	3.895	0.875	49.15	11.54–476.03	<0.001
Lateral LN metastasis	2.632	0.498	13.90	5.47–38.98	<0.001

β, regression coefficient; SE, standard error; OR, odds ratio; CI, confidence interval; LN, lymph node. Estimates are from Firth penalized-likelihood logistic regression; 95% CIs are profile penalized-likelihood intervals and P values are from penalized likelihood-ratio tests, so that the intervals and the P values derive from the same profile penalized likelihood and always agree. Firth’s method was used because only one of the 166 patients without central LN metastasis had LN-prRLN metastasis, which makes the ordinary maximum-likelihood estimate for that variable upwardly biased and very imprecise; complete and quasi-complete separation were excluded, all coefficients being finite and no fitted probability reaching 0 or 1. The upper panel shows the full six-variable model (intercept, −6.903); after non-significant capsular invasion was removed, the model was refitted in the five remaining variables (lower panel) to build the nomogram (final-model intercept, −6.963).

After capsular invasion was removed, the model was refitted in the five remaining variables by the same penalized-likelihood method to obtain the final model underlying the nomogram (**Table 3**, lower panel); the adjusted odds ratios were 4.46 for multifocality, 2.87 for tumor diameter >10 mm, 6.28 for isthmic/near-isthmic location, 49.15 for central lymph node metastasis and 13.90 for lateral lymph node metastasis (all P ≤ 0.013). The two panels agree in the direction and significance of every retained predictor, the small numerical differences reflecting only re-estimation once capsular invasion was dropped. The five independent predictors were used to construct the nomogram (**Figure 1**). On the points scale, central lymph node metastasis contributed the greatest weight (100 points), followed by lateral lymph node metastasis, isthmic/near-isthmic location, multifocality and tumor diameter >10 mm. For an individual patient, the points assigned to each present risk factor are summed to obtain the total points, which map to the predicted probability of LN-prRLN metastasis. The corresponding penalized logistic model was: logit(P) = −6.96 + 1.50 × (multifocality) + 1.05 × (tumor >10 mm) + 1.84 × (isthmic/near-isthmic location) + 3.90 × (central lymph node metastasis) + 2.63 × (lateral lymph node metastasis), where each variable is coded 1 if present and 0 if absent.

**Figure 1:**
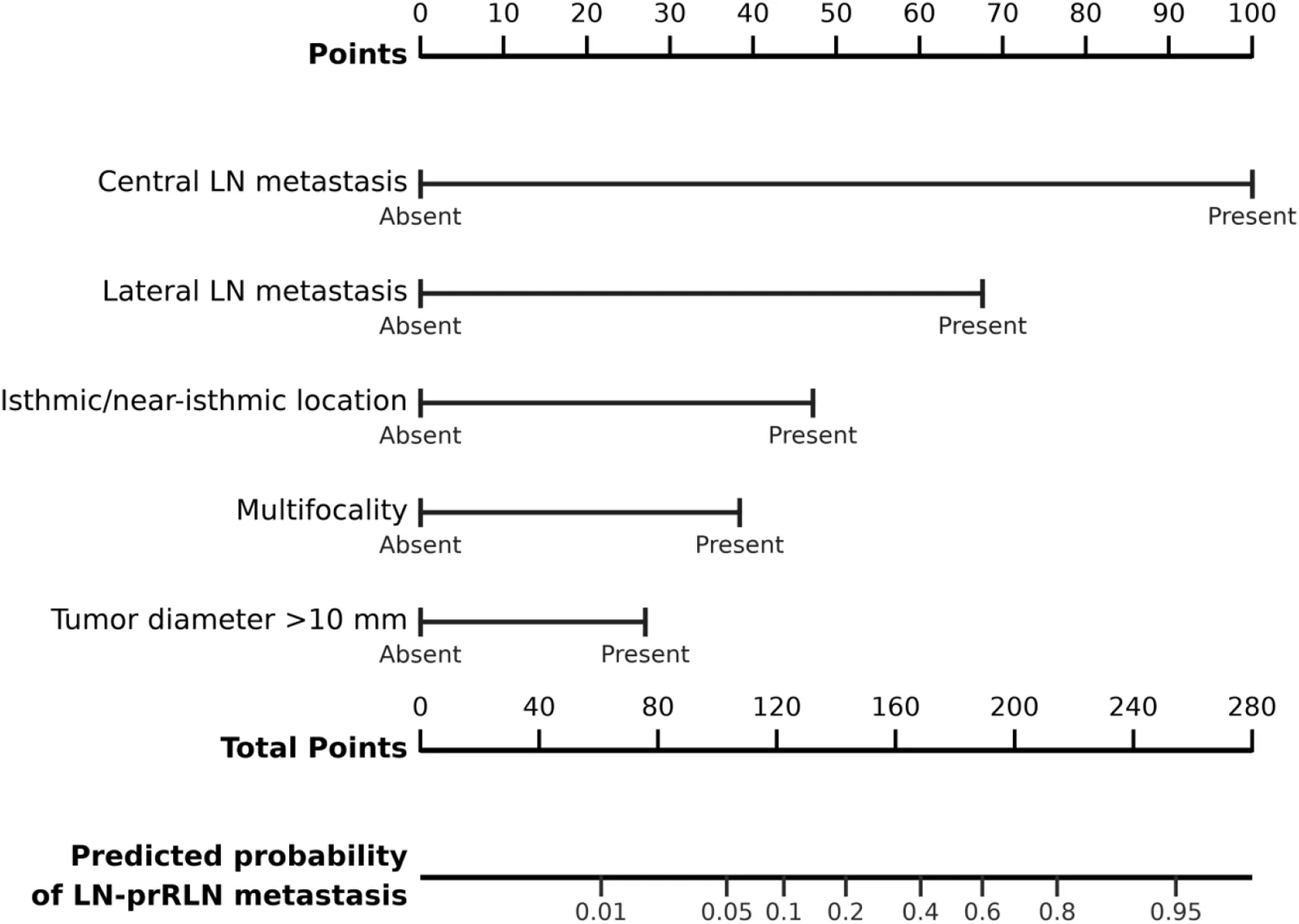
Nomogram for predicting the probability of LN-prRLN metastasis in papillary thyroid carcinoma. For each patient, a vertical line is drawn from the value of each predictor to the Points axis; the points for all five predictors are summed to obtain Total Points, which are projected onto the bottom axis to read the predicted probability of LN-prRLN metastasis.

### Discrimination

The nomogram discriminated well between patients with and without LN-prRLN metastasis, with an AUC of 0.936 (95% CI 0.901–0.971, DeLong method) (**Figure 2**). After 1,000-sample bootstrap internal validation in which the whole model-building procedure was repeated within every resample, the optimism was 0.009 and the optimism-corrected C-statistic 0.927, indicating little overfitting. At the optimal threshold probability of 0.226 identified by the maximum Youden index (0.739), the model achieved a sensitivity of 0.843 and a specificity of 0.896, detecting 43 of the 51 metastases with 28 false positives (positive predictive value 0.606, negative predictive value 0.968). Because leaving an involved node behind is the more costly error, a lower threshold may be preferable in practice: at a predicted probability of 0.044 sensitivity rose to 0.980 while 49.4% of the cohort was still classified as low risk. When each predictor was evaluated individually (**Figure 3**, **Table 4**), all had an AUC significantly greater than 0.5. Central lymph node metastasis had the highest single-variable discrimination (AUC 0.797, 95% CI 0.761–0.831), followed by lateral lymph node metastasis (AUC 0.726, 95% CI 0.657–0.797), multifocality (AUC 0.663, 95% CI 0.593–0.726), tumor diameter >10 mm (AUC 0.660, 95% CI 0.588–0.729) and isthmic/near-isthmic location (AUC 0.567, 95% CI 0.508–0.628; P = 0.029). On DeLong’s test for correlated curves the combined nomogram discriminated significantly better than every individual predictor, including central lymph node metastasis (all P<0.001).

**Figure 2:**
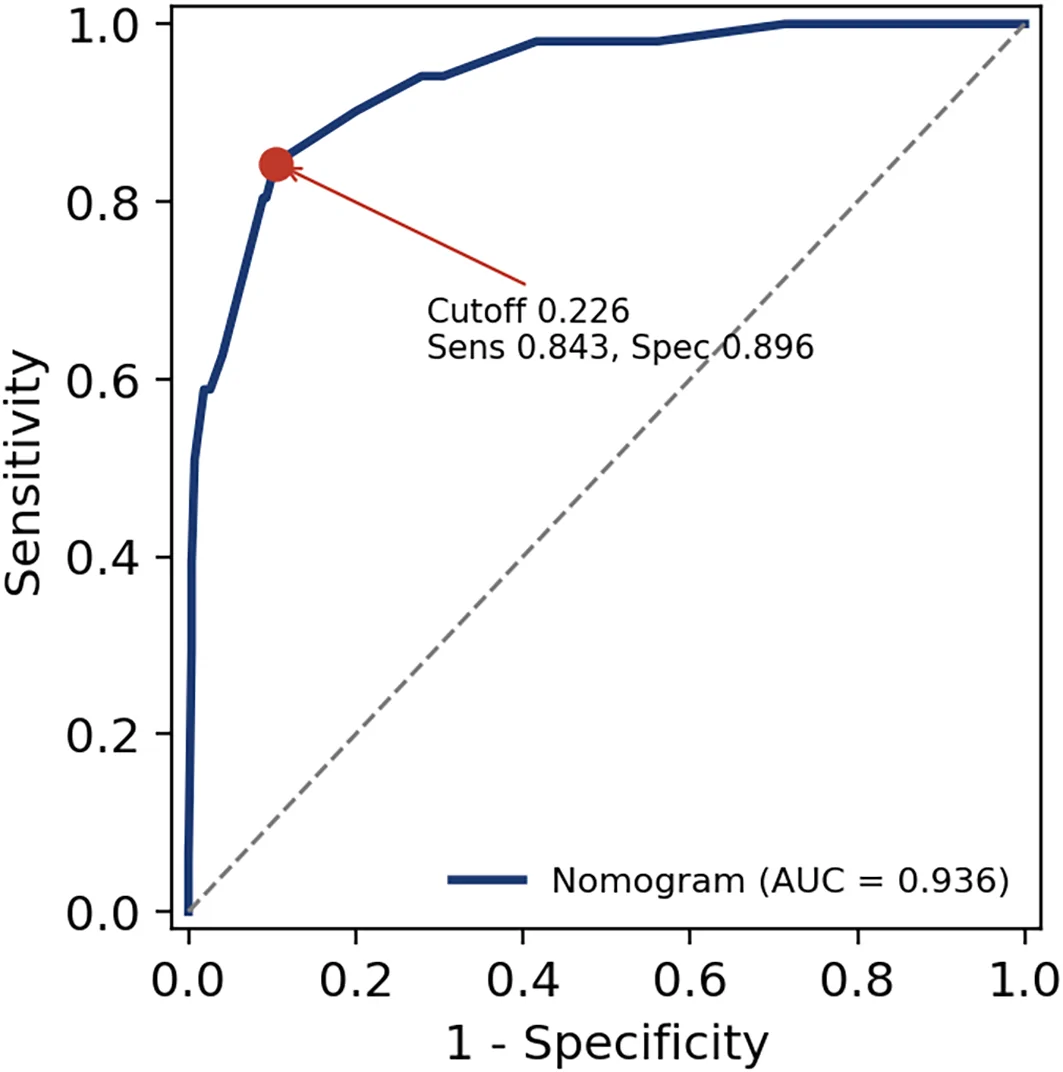
Receiver operating characteristic (ROC) curve of the nomogram for predicting LN-prRLN metastasis. The area under the curve was 0.936 (95% CI 0.901–0.971, DeLong method). The marked point corresponds to the optimal cutoff (0.226; sensitivity 0.843, specificity 0.896).

**Figure 3:**
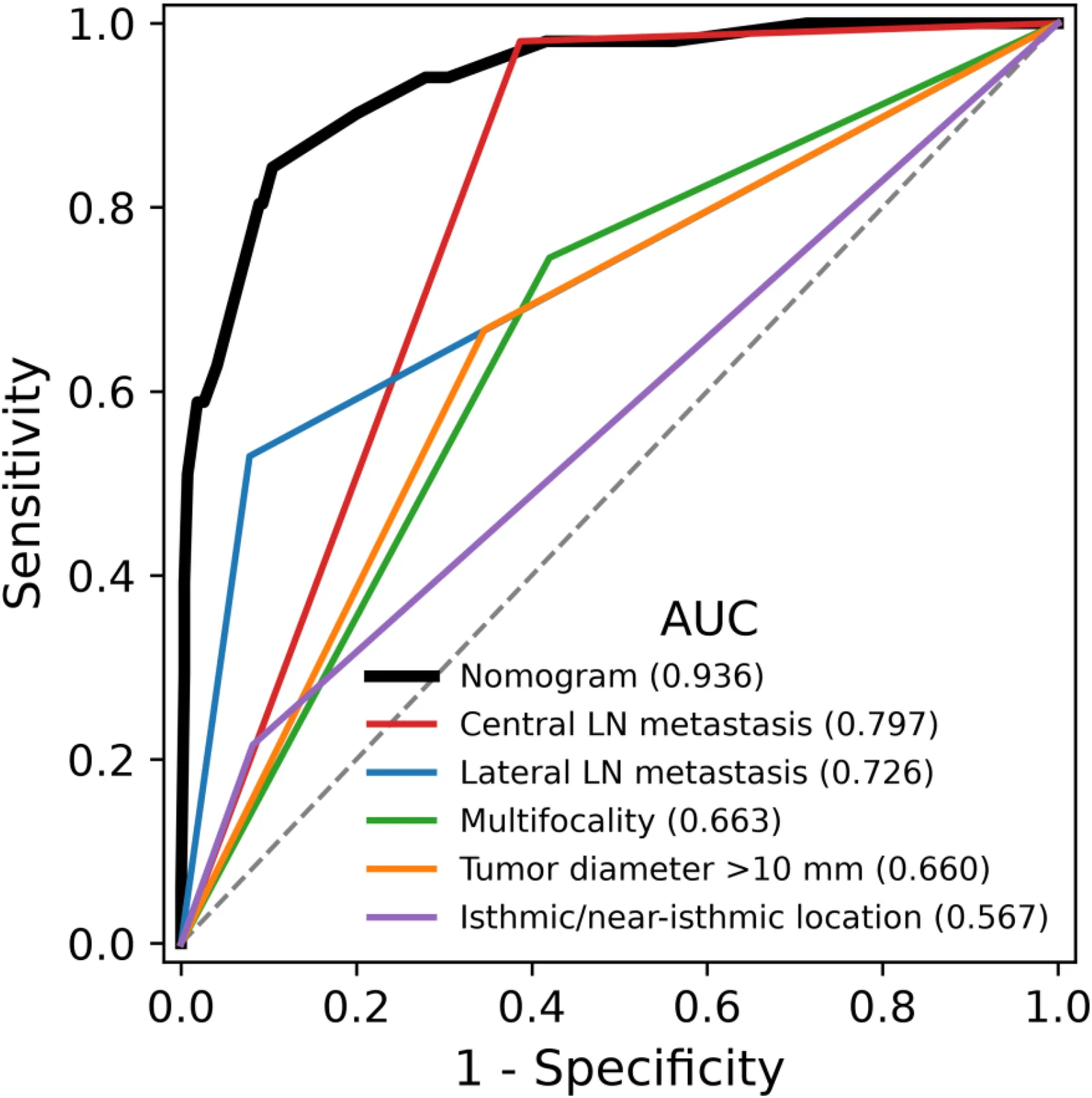
Comparison of ROC curves for the combined nomogram and each individual predictor. The combined nomogram (AUC 0.936) outperformed central lymph node metastasis (0.797), lateral lymph node metastasis (0.726), multifocality (0.663), tumor diameter >10 mm (0.660) and isthmic/near-isthmic location (0.567).

**Table 4:** Discriminative performance of the nomogram and individual predictors for LN-prRLN metastasis.

Variable	AUC	95% CI	Sensitivity	Specificity	P value
Nomogram (combined)	0.936	0.901–0.971	0.843	0.896	<0.001
Central LN metastasis	0.797	0.761–0.831	0.980	0.613	<0.001
Lateral LN metastasis	0.726	0.657–0.797	0.529	0.922	<0.001
Multifocality	0.663	0.593–0.726	0.745	0.580	<0.001
Tumor diameter >10 mm	0.660	0.588–0.729	0.667	0.654	<0.001
Isthmic/near-isthmic location	0.567	0.508–0.628	0.216	0.918	0.029

AUC, area under the receiver operating characteristic curve; CI, confidence interval; LN, lymph node. Confidence intervals were obtained by DeLong’s method and the tabulated P values test the null hypothesis that the AUC equals 0.5. For the nomogram, the optimal cutoff (0.226) was defined by the maximum Youden index (0.739); at this cutoff the positive and negative predictive values were 0.606 and 0.968. The nomogram discriminated significantly better than each individual predictor on DeLong’s test for correlated curves (all P<0.001). For the five binary predictors the tabulated sensitivity and specificity are those of the variable itself, which admits only one cutoff.

### Calibration

Internal validation was performed with 1,000 bootstrap resamples. The apparent calibration slope was 1.071, reflecting the shrinkage already imposed by the penalized fit; after optimism correction the slope was 0.961 and the calibration intercept −0.024, both close to their ideal values of 1 and 0 and indicating only slight optimism in the regression coefficients. The apparent and bias-corrected calibration curves both lay close to the ideal 45-degree line across the range of predicted probabilities, with only modest deviation at intermediate-to-high predicted risk (**Figure 4**). The Hosmer–Lemeshow test showed no significant lack of fit (χ² = 9.72, df = 8; P = 0.285), although with only 27 distinct predicted probabilities the required grouping is not unique, and this result should not be over-interpreted. Internal calibration was therefore good, though the agreement between predicted and observed probabilities still needs to be checked in an independent cohort.

**Figure 4:**
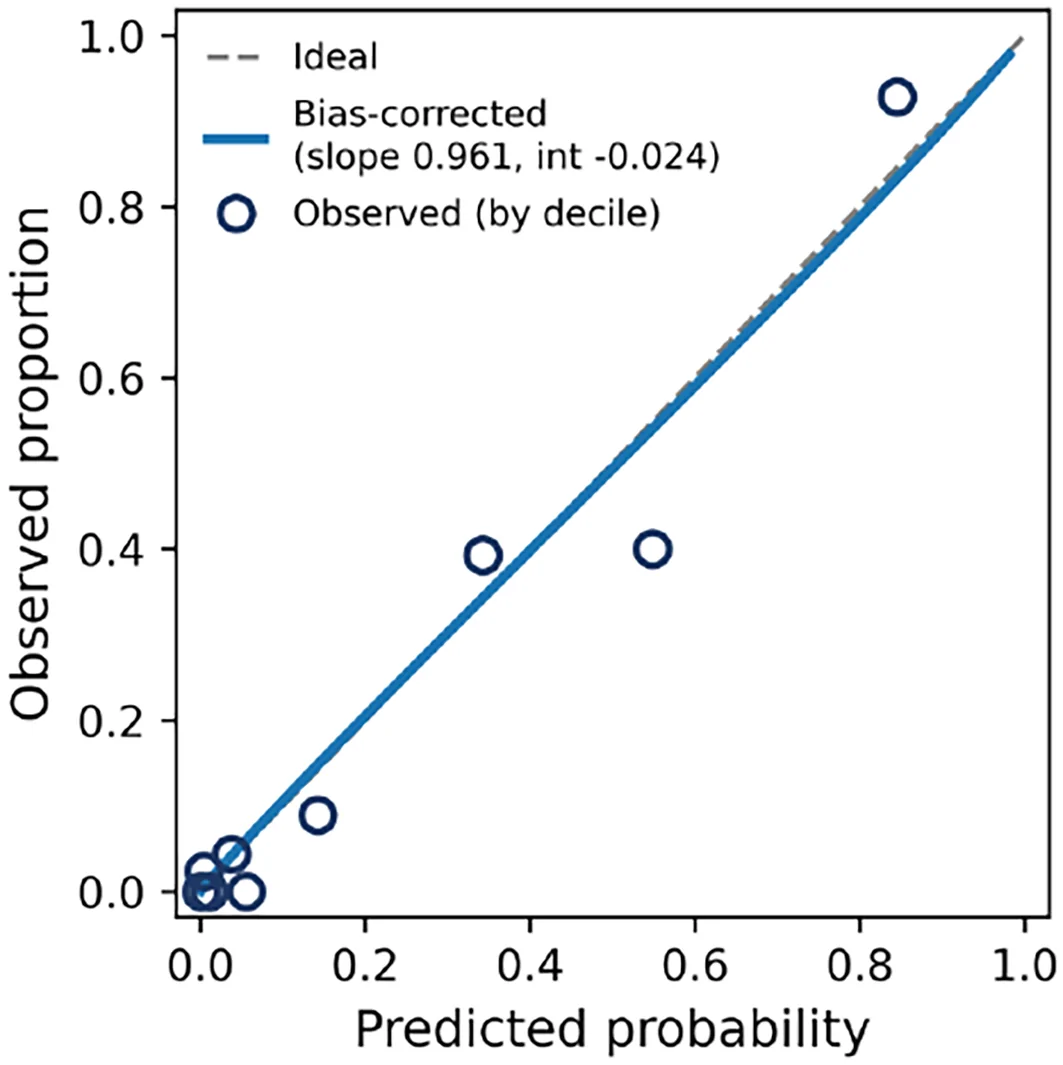
Calibration of the nomogram based on 1,000 bootstrap resamples. The dashed line represents ideal calibration; the bias-corrected calibration line (blue; optimism-corrected slope 0.961, intercept −0.024) closely approximates it, and open circles denote observed versus predicted probabilities grouped by decile.

### Clinical utility

Decision curve analysis was used to assess the clinical net benefit of the nomogram (**Figure 5**). The nomogram yielded a higher net benefit than both the treat-all and treat-none strategies across essentially the whole range of high-risk threshold probabilities examined (approximately 0.01–0.95). To express this in clinically interpretable terms, at a threshold probability of 0.10, the point at which a surgeon would accept nine unnecessary LN-prRLN dissections in order to avoid leaving one metastatic node behind, using the nomogram rather than dissecting every patient would spare about 52 dissections per 100 patients for the same number of metastases detected; at a threshold of 0.20 the corresponding figure is about 65 per 100. These are apparent values obtained in the development cohort, so both the preferred threshold and the size of the benefit require prospective confirmation.

**Figure 5:**
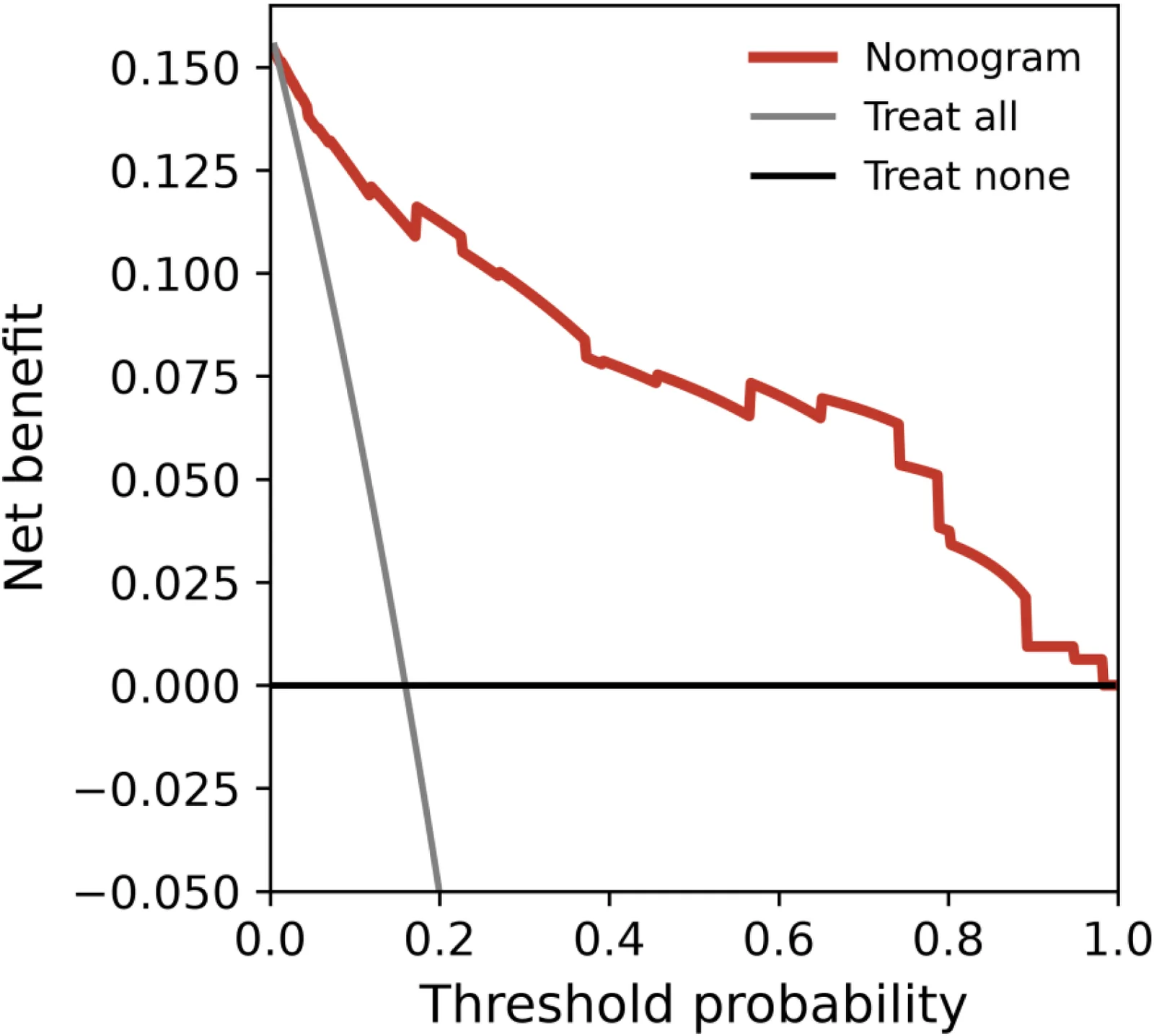
Decision curve analysis of the nomogram. The x-axis shows the high-risk threshold probability and the y-axis the net benefit. Across approximately 0.01–0.95, the nomogram (red) provided greater net benefit than the treat-all (gray) and treat-none (black) strategies.

### Sensitivity analyses and selection stability

Substituting the 55-year age threshold of the 8th-edition staging system for the 45-year threshold did not change the findings: age <55 years was not associated with LN-prRLN metastasis on univariable analysis (OR 1.39, 95% CI 0.56–3.46; P = 0.482) and remained non-significant when added to the final model (OR 1.31, 95% CI 0.44–4.34; P = 0.642), as did age <45 years (OR 1.40; P = 0.479) and male sex (OR 1.30; P = 0.565). Replacing the >10 mm dichotomy with maximum tumor diameter as a continuous variable left discrimination essentially unchanged (AUC 0.933, 95% CI 0.895–0.970) but the size effect was no longer significant after adjustment (OR 1.53 per 1 cm, 95% CI 0.92–2.56; P = 0.103), so the clinically conventional dichotomy was retained. Removing central nodal status reduced the AUC from 0.936 to 0.851 (95% CI 0.788–0.914; DeLong P<0.001). Variance inflation factors were all below 1.07, giving no indication of collinearity among the five predictors. Across the 1,000 bootstrap resamples the retained predictors survived the full selection procedure in 100% (lateral lymph node metastasis), 98% (central lymph node metastasis), 94% (multifocality), 70% (isthmic/near-isthmic location) and 69% (tumor diameter >10 mm) of samples, whereas capsular invasion survived in only 20% and age and sex in fewer than 7%.

## Discussion

In this series of 320 patients with PTC, LN-prRLN metastasis occurred in 15.9% of cases, well within the 2.7–38.3% range reported previously (4, 6, 7). Five variables emerged as independent predictors (multifocality, tumor diameter >10 mm, isthmic/near-isthmic location, central lymph node metastasis and lateral lymph node metastasis), and a nomogram built from them discriminated well (AUC 0.936), calibrated acceptably, and added net benefit on decision curve analysis. Each of these predictors was coded from the final histopathological report, but each has a preoperative counterpart that is available when the operation is planned: tumor size, focality and isthmic location are documented by routine preoperative ultrasound, and central and lateral nodal status — the two strongest predictors — are assessed before surgery by ultrasonography and contrast-enhanced computed tomography, both of which are performed routinely in every patient with thyroid cancer at our center and which together substantially improve preoperative nodal staging; this is the same information on which decisions about central and lateral neck dissection are based in everyday practice. The nomogram is therefore intended to be used with these preoperative, imaging-based assessments, to help plan the extent of central neck dissection and to judge whether the dissection should be carried into the deep right paratracheal compartment. Because the model was derived against pathological rather than imaging-based nodal status, however, the performance reported here is an upper bound on what can be expected in that setting, and we have qualified the claimed clinical use accordingly. We wish to state this limitation explicitly, because it is the principal constraint on the clinical applicability of the nomogram. Central and lateral nodal status in this study were encoded from the final histopathological report — that is, from information that exists only after the operation the model is intended to help plan — and preoperative imaging recovers that information only imperfectly. Ultrasonography, the cornerstone of preoperative nodal assessment, identifies only about one-third to one-half of pathologically proven nodal metastases, and its sensitivity is lowest in the central compartment, where the overlying thyroid gland obscures the nodes; adding contrast-enhanced computed tomography improves detection, but the combined assessment still misses roughly half of central compartment metastases (26, 27). The discrimination and calibration reported here therefore describe a pathology-based version of the model rather than the imaging-based version that would be used in practice, and performance will be attenuated — to a degree that cannot be measured with the present data — once imaging-derived nodal status is substituted for the pathological standard.

Central lymph node metastasis carried by far the greatest weight in the model. This fits the anterograde, stepwise spread that is typical of PTC, in which the LN-prRLN behaves as a deeper extension of the central compartment and is usually involved only after the superficial central nodes (24); pooled data tell the same story, with one meta-analysis reporting an odds ratio of 22.23 (95% CI 16.17–30.56) between superficial central metastasis and LN-prRLN involvement (7). The size of the odds ratio in our own data, however, calls for caution. Only one of the 166 patients without central metastasis had LN-prRLN metastasis. These data are sparse rather than separated: ordinary maximum likelihood still converged, with all coefficients finite and no fitted probability at 0 or 1, but the estimate it returned (OR 76.52) carries the upward small-sample bias expected of a cell containing a single event. Fitting the multivariable model by Firth’s penalized-likelihood method reduced this to a more stable OR of 44.52, although the profile penalized-likelihood interval remained wide (10.59–426.16). In other words, the association is real and clinically intuitive, but its exact magnitude is not; larger external cohorts remain essential to establish it precisely.

That single patient is worth dwelling on, because it is also an example of skip metastasis. Of the 166 patients with no superficial central metastasis, this one (1 of 166, 0.6%) still had LN-prRLN metastasis, and it was the only node-positive case in the whole series (1 of 51, 2.0%) arising in the setting of a negative central compartment. The primary was a 4-mm multifocal carcinoma of the lower pole; both the superficial central and the lateral compartments were clear on pathology, leaving the LN-prRLN as the only site of disease (**Table 5**). Skip metastasis, in which spread bypasses the first-echelon central compartment, is an uncommon but well-described pattern in PTC and is usually ascribed to direct or aberrant lymphatic channels draining the tumor (25). Our case carries the idea over to the LN-prRLN and shows that the deep right paratracheal nodes can occasionally be involved while the superficial central nodes stay clear. It is also the single event responsible for the sparse-data bias and the inflated maximum-likelihood odds ratio noted above. The practical message is that a negative central compartment lowers the odds of LN-prRLN metastasis sharply but does not abolish them: the empirical floor here was 1 in 166, or about 0.6%, low but not zero. Some caution is therefore still warranted, especially in multifocal tumors, since an occult deep node left behind in an apparently low-risk patient could seed persistent or recurrent central disease. Because this rests on a single case, we regard it as hypothesis-generating rather than conclusive.

**Table 5:** Distribution of LN-prRLN metastasis according to combined central and lateral lymph node status.

Central LN status	Lateral LN status	Patients, n	LN-prRLN +, n (%)	LN-prRLN −, n
Negative	Negative	152	1 (0.7)	151
Negative	Positive	14	0 (0.0)	14
Positive	Negative	120	23 (19.2)	97
Positive	Positive	34	27 (79.4)	7
Total	—	320	51 (15.9)	269

LN, lymph node; LN-prRLN, lymph nodes posterior to the right recurrent laryngeal nerve. The rate of LN-prRLN metastasis rose steeply with central and lateral nodal burden, from 0.7% when both compartments were negative to 79.4% when both were positive; because the central-negative, lateral-positive stratum contained only 14 patients and no events, the gradient is not strictly monotonic across all four strata. The single central-node-negative patient with LN-prRLN metastasis (Negative/Negative row) represents an isolated skip metastasis and is the single event that makes the ordinary maximum-likelihood odds ratio for central lymph node metastasis upwardly biased and imprecise. Percentages for LN-prRLN + are within-row; the total row percentage is of the whole cohort.

Lateral lymph node metastasis ranked next. It marks a heavier nodal burden and wider lymphatic dissemination, and its link with LN-prRLN metastasis has been noted repeatedly (7, 8); in practice, a patient with proven or strongly suspected lateral neck disease deserves close attention to the deep right paratracheal region and, where appropriate, its clearance. Multifocality and larger tumor size are long-recognized markers of biologically aggressive PTC and of central nodal metastasis more generally (2, 3), and their independent effect on LN-prRLN risk here agrees with earlier meta-analyses, which reported pooled odds ratios of 2.08 for multifocality and 3.40 for tumors larger than 10 mm (7, 8).

An isthmic or near-isthmic location was also independently predictive and carried one of the larger adjusted odds ratios in the model. Isthmic tumors drain in a distinctive, often bidirectional way and have been tied to higher rates of central metastasis and to less predictable nodal spread than tumors elsewhere in the gland (13). Our data carry that observation over to the LN-prRLN specifically: an isthmic primary should raise suspicion for deep right paratracheal disease. Capsular invasion was significant on univariable testing but dropped out after adjustment, most likely because its effect is already captured by the correlated and more strongly predictive nodal variables.

Set against the models already published for this subregion, our results are reassuring in some respects and discordant in others. Luo et al. retained age, tumor size, capsular invasion and right lateral nodal metastasis and reached an AUC of 0.790 in derivation and 0.878 in a split validation sample (9); we reproduce the effects of tumor size and lateral nodal metastasis but not those of age or, after adjustment, capsular invasion. Zhu et al. and Deng et al. both found the burden of disease in the superficial central subgroups, namely the prelaryngeal, pretracheal and paratracheal nodes, to be the dominant signal (10, 11), which is consistent with the very large weight carried by superficial central metastasis in our model, although we treated that compartment as a single binary variable rather than counting metastatic nodes by subgroup. Gong et al. found sonographic features of the primary tumor and metastasis in the nodes anterior to the right RLN to be independently predictive (4), variables we did not record. The apparent discrimination of our model (0.936) is higher than that of these earlier models, but the comparison is not like for like: our two strongest predictors are pathological rather than imaging-based, which inflates apparent performance relative to models restricted to preoperatively available information, and no model, ours included, has yet been tested in an external cohort. The practical conclusion is not that one model is better than another, but that tumor size, multifocality and the burden of superficial central and lateral nodal disease recur across independent cohorts, whereas age, sex and capsular invasion do not. Against this background, the present study contributes three things the literature still lacks. First, it restricts itself to five variables documented in every routine work-up, making the model transportable to centers that do not record nodal subgroup counts or contrast-enhanced ultrasound features. Second, it provides the most rigorous internal evaluation in this literature to date — penalized-likelihood estimation for sparse data, bootstrap validation that repeats the entire variable-selection procedure, and TRIPOD-compliant reporting with a public analysis script — giving future external validations a stable, reproducible reference. Third, it quantifies the risk gradient within the central-node-positive group (from 19% to 79% according to lateral status; **Table 5**) and the residual risk when both compartments are clear (about 0.6%), neither of which earlier models reported.

One discrepancy deserves comment. The meta-analysis from which we take pooled effect sizes reported male sex (OR 1.47) and age no greater than 45 years (OR 1.63) as significant risk factors for LN-prRLN metastasis (7), yet neither was associated with the outcome in our cohort, alone or after adjustment. The most likely explanation is limited power combined with limited contrast: with 51 events this study is well powered only for large effects, and the cohort was young (mean age 40.4 years, 70.9% under 45), which leaves little room for an age gradient to declare itself. Substituting the 55-year threshold of the 8th-edition staging system did not alter this. We therefore regard the absence of age and sex from our nomogram as a feature of this cohort rather than as evidence against their relevance, and we would not argue from these data that they should be dropped from future models.

What the nomogram really offers is a way to make an intuitive intraoperative judgment explicit and quantitative. Combining preoperative features (tumor size, focality, location) with imaging-based nodal findings (central and lateral status), the surgeon can put an individual probability on LN-prRLN metastasis and set it against the added risk of RLN traction injury and parathyroid devascularization that clearing this compartment entails (4, 7). At the cutoff we identified (predicted probability 0.226), the model caught most patients with metastasis (sensitivity 0.843) while sparing most of those without it (specificity 0.896), and decision curve analysis pointed to a favorable trade-off over a broad range of plausible thresholds.

Because superficial central metastasis dominates the model and occupies the immediately adjacent tier of the same compartment, it is closely tied to LN-prRLN status rather than being a mechanistically independent marker, and the nomogram must therefore justify itself against central nodal status used alone. It does so on two counts. First, central status by itself discriminated only moderately (AUC 0.797), whereas adding tumor size, focality, isthmic location and lateral nodal status raised the AUC to 0.936 (DeLong P<0.001; **Table 4**), and the five predictors were only weakly correlated with one another (all variance inflation factors below 1.07). Second, and more usefully, the gain represents genuine re-stratification within the central-positive group, only 32% of whom (50 of 154) harbored LN-prRLN metastasis: the observed rate rose from 19% (23 of 120) when the lateral neck was negative to 79% (27 of 34) when it was positive (**Table 5**). A graded probability therefore separates patients whom a binary central-node trigger would treat identically, and at the opposite extreme it identifies a risk of roughly 1 in 166, about 0.6%, when the superficial central compartment is clear. Predictions in that stratum nevertheless rest on a single event and should not be used to justify dissection on their own.

The study has several strengths: a reasonably large, consecutively collected single-center cohort; LN-prRLN specimens that were dissected and labeled separately; complete data; a sample size that exceeds the recommended minimum for developing a five-predictor model (15); and an evaluation covering discrimination, optimism-corrected bootstrap calibration and decision curve analysis, reported according to the TRIPOD framework (23). Its limitations are equally clear. The study was retrospective and confined to one center, and the nomogram was validated only internally, so external, preferably multicenter and prospective, validation is essential before clinical use. Predictors were selected by univariable screening at P<0.05, which is no longer the recommended strategy for prediction-model development because it inflates apparent performance; we retained it for comparability with the existing LN-prRLN literature and quantified its cost by repeating the entire selection procedure within every bootstrap sample, which is also why the two weaker predictors survived selection in only about 70% of resamples. The moderate selection frequencies of these two variables identify them as the least stable members of the final set, and they should be interpreted as secondary adjusters rather than core predictors: their adjusted odds ratios are modest and imprecise (95% CIs 1.97–21.20 and 1.25–6.83, respectively). The optimism-corrected performance estimates already penalize this instability, because the full selection procedure — including the possible exclusion of these two variables — was repeated within every bootstrap resample. Whether they belong in the model at all is a question that external validation will answer better than any internal procedure. The central-node-negative stratum contained a single event, which leaves the odds ratio for central nodal status imprecise; Firth penalized-likelihood estimation reduced but did not remove this imprecision, and with only 51 events the number of predictors that can be estimated reliably is limited. Central and lateral nodal status — the two strongest predictors — were coded from final pathology, whereas in clinical practice they are assessed by preoperative imaging; although every patient at our center undergoes both ultrasound and contrast-enhanced CT, which substantially improves preoperative nodal staging, imaging may still understage the central compartment in a minority of patients, so the discrimination and calibration reported here remain optimistic relative to preoperative application, and quantifying performance with imaging-derived inputs is a priority for the planned prospective validation. We stress this point because it currently limits how the nomogram may legitimately be used. The two predictors that carry most of its discriminative power are, as coded, postoperative facts rather than preoperative measurements, and the imaging modalities available to approximate them are known to understage the central compartment in a substantial proportion of patients, particularly when metastatic deposits are small (26, 27). Some misclassification of nodal status in real preoperative use is therefore unavoidable, and the apparent AUC of 0.936 should be read as an optimistic upper bound rather than as the performance achievable in the clinic. Until the model is refitted and validated with imaging-coded predictors in a prospective cohort, it should be used to structure — not to replace — clinical judgment, and a low predicted probability should not, by itself, be taken as sufficient grounds to omit dissection of the deep right paratracheal compartment. Because LN-prRLN dissection was routine at our center, the cohort is not selected with respect to the dissection decision itself; generalizability is nonetheless limited by the single-center setting and by restriction to the right side, where this compartment exists, and practice patterns that differ elsewhere may affect transportability. We also did not include molecular markers such as BRAF status or quantitative ultrasound features. Multicenter prospective validation, the addition of imaging and molecular data, and Bayesian modeling to stabilize the estimates for sparsely observed predictors are all natural next steps.

## Conclusion

Multifocality, tumor diameter >10 mm, isthmic/near-isthmic location, and central and lateral lymph node metastasis are independent predictors of LN-prRLN metastasis in PTC. A nomogram built from these five factors discriminated well, calibrated acceptably, and showed a positive net benefit in the development cohort, and it may help surgeons make individualized, risk-adapted decisions about LN-prRLN dissection. Its inputs are, in practice, obtainable before surgery — tumor size, focality and location from routine ultrasound, and central and lateral nodal status from the preoperative ultrasound and contrast-enhanced CT that every patient at our center undergoes — so the nomogram is intended to support the planning of central neck dissection; but because it was derived against pathological nodal status, its performance with imaging-based inputs will be attenuated, and it should be treated as a decision-support tool pending external and prospective validation. In particular, because central and lateral nodal status were coded from the final histopathological report, and because preoperative ultrasound and contrast-enhanced computed tomography detect central compartment metastasis with a sensitivity far below the accuracy of pathological diagnosis (26, 27), the nomogram must not be applied as though these nodal inputs were known with certainty before surgery; its reported performance is an upper bound on preoperative performance, and prospective validation with imaging-coded predictors is a prerequisite for routine clinical use.

## Data Availability

The datasets presented in this article are not readily available because We retrospectively reviewed 320 patients with papillary thyroid carcinoma (PTC) treated at a single tertiary center. Requests to access the datasets should be directed to Qiran Lu, 1063671621@qq.com.
